# Habitats generated by the restoration of coal mining subsidence land differentially alter the content and composition of soil organic carbon

**DOI:** 10.1371/journal.pone.0282014

**Published:** 2023-02-21

**Authors:** Yongkang Zhao, Guodong Zheng, Huaizhi Bo, Yijing Wang, Junyu Dong, Changchao Li, Yan Wang, Shuwan Yan, Kang Liu, Zhiliang Wang, Jian Liu

**Affiliations:** 1 Environment Research Institute, Shandong University, Qingdao, China; 2 Lunan Geo-engineering Exploration Institute, Jining, China; Thapar Institute of Engineering and Technology, INDIA

## Abstract

The content and composition of soil organic carbon (SOC) can characterize soil carbon storage capacity, which varies significantly between habitats. Ecological restoration in coal mining subsidence land forms a variety of habitats, which are ideal to study the effects of habitats on SOC storage capacity. Based on the analysis of the content and composition of SOC in three habitats (farmland, wetland and lakeside grassland) generated by different restoration time of the farmland which was destroyed by coal mining subsidence, we found that farmland had the highest SOC storage capacity among the three habitats. Both dissolved organic carbon (DOC) and heavy fraction organic carbon (HFOC) exhibited higher concentrations in the farmland (20.29 mg/kg, 6.96 mg/g) than in the wetland (19.62 mg/kg, 2.47 mg/g) or lakeside grassland (5.68 mg/kg, 2.31 mg/g), and the concentrations increased significantly over time, owing to the higher content of nitrogen in the farmland. The wetland and lakeside grassland needed more time than the farmland to recover the SOC storage capacity. The findings illustrate that the SOC storage capacity of farmland destroyed by coal mining subsidence could be restored through ecological restoration and indicate that the recovery rate depends on the reconstructed habitat types, among which farmland shows great advantages mainly due to the nitrogen addition.

## Introduction

Soil is the largest terrestrial organic carbon (OC) pool and so even slight changes in soil may have a large impact on atmospheric carbon dioxide concentrations [1, 2]. Land use/cover change is one of the main factors affecting soil organic carbon (SOC) storage capacity [3–6], which may even determine whether soil is a carbon source or sink [7]. While restoration of soil can recover its SOC storage capacity over time [8–10], it is necessary to investigate the mechanisms of SOC storage in different habitats to take effective measures to protect existing SOC pools or to recover SOC storage capacity.

Land use/cover change produces different habitats that are the driving factors of terrestrial carbon sinks [11]. Conventional tillage of agricultural soils, which causes great soil disturbance and provides little mulch, enhances respiration of topsoil and leads to losses of SOC, soil degradation and decline in agricultural sustainability [12]. Some studies have shown that improved agricultural practices, such as no-tillage agriculture, have great potential to increase the content of OC sequestered in soils [10, 12–15]. Wetlands are carbon sinks or become carbon sources when land use/cover change [7], but it is undeniable that wetlands can accumulate large quantities of SOC [16]. Besides, the contribution of grasslands to SOC sequestration is also notable [17–19]. In the past, studies about SOC have mostly focused on the content and composition of SOC in original habitats, while few studies have been conducted on soils in different habitats generated by the restoration of the coal mining subsidence.

China is the largest coal-producing country in the world, with coal still playing an irreplaceable role in the nation’s energy supply [20]. At the same time, coal mining has a series of consequences on the ecological environment, such as eliminating vegetation and altering soil [21–24]. Underground coal mining has caused subsidence of farmland and other habitats, resulting in serious damage to ecosystems in coal mining areas [21, 25–27]. Vegetation restoration is the normal method of ecological reconstruction in disturbed areas, and can be achieved by both active interventions and natural regeneration methods [25, 28]. Recent studies about coal mining subsidence have focused on how revegetation type and microorganisms affected SOC [29, 30]. Insufficient attention has been paid to the differences of SOC content and composition between habitats generated by the restoration of coal mining subsidence land, which are important both for land management and responses to environmental disturbances [31].

In this study, samples were gathered from 30 sites at two parks formed by the restoration of coal mining subsidence with different restoration times. To explore the impact of different habitats on SOC, we divided SOC in different forms to better describe the changes of soil organic carbon. We additionally quantified soil properties of three habitats (farmland, wetland and lakeside grassland). We hypothesized that the SOC storage function of farmland destroyed by coal mining subsidence could be restored through ecological restoration and that the recovery rate would depend on the reconstructed habitat type.

## Materials and methods

### Ethics statement

We collected soil for our study with the official permission of the Management Committee of Jining Constructed Wetland for the permission of sample collection. There is no endangered or protected plant species being destroyed.

### Study area and field sampling

Shandong Zoucheng Taiping National Wetland Park (ZWP, 116°47’05” ~ 116°50’13” E, 35°24’51” ~ 35°26’12” N), built in 2017, is located in the west of Zoucheng City, Shandong Province, which is a typical coal mining subsidence area in the North China Plain. ZWP has a catchment area of 10.02 km^2^. The Shili Lake Ecological Wetland Park (JWP, 116°39’33” ~ 116°40’16” E, 35°22’11” ~ 35°23’2” N), built in 2019, is located in Jining, Shandong Province. JWP has a catchment area of 2.06 km^2^. The three habitats were generated by the restoration of the farmland which was destroyed by the coal mining subsidence. Specifically, the areas which were less affected by the coal mining subsidence were restored to the farmland, and wetlands were those areas which have been standing water for a long time. Lakeside grasslands were generated by the accumulation of soil obtained through excavating the surface layer of the part that was heavily affected by the coal mining subsidence. In these two areas, the main soil types are hydromorphic soils, with poor permeability but high soil fertility. The mean annual temperature is 14.2°C and the mean annual precipitation is 743 mm. The main vegetation types in wetlands are *Typha orientalis* C. Presl and *Phragmites australis* (Cav.) Trin. ex Steud. The main crop grown in farmlands is *Zea mays* L. The tillage managements in both parks are no-tillage and crop residues were left on the soil surfaces. The lakeside grasslands are dominated by *Setaria viridis* (L.) P. Beauv. and *Liriope spicata* Lour.

**Fig 1:**
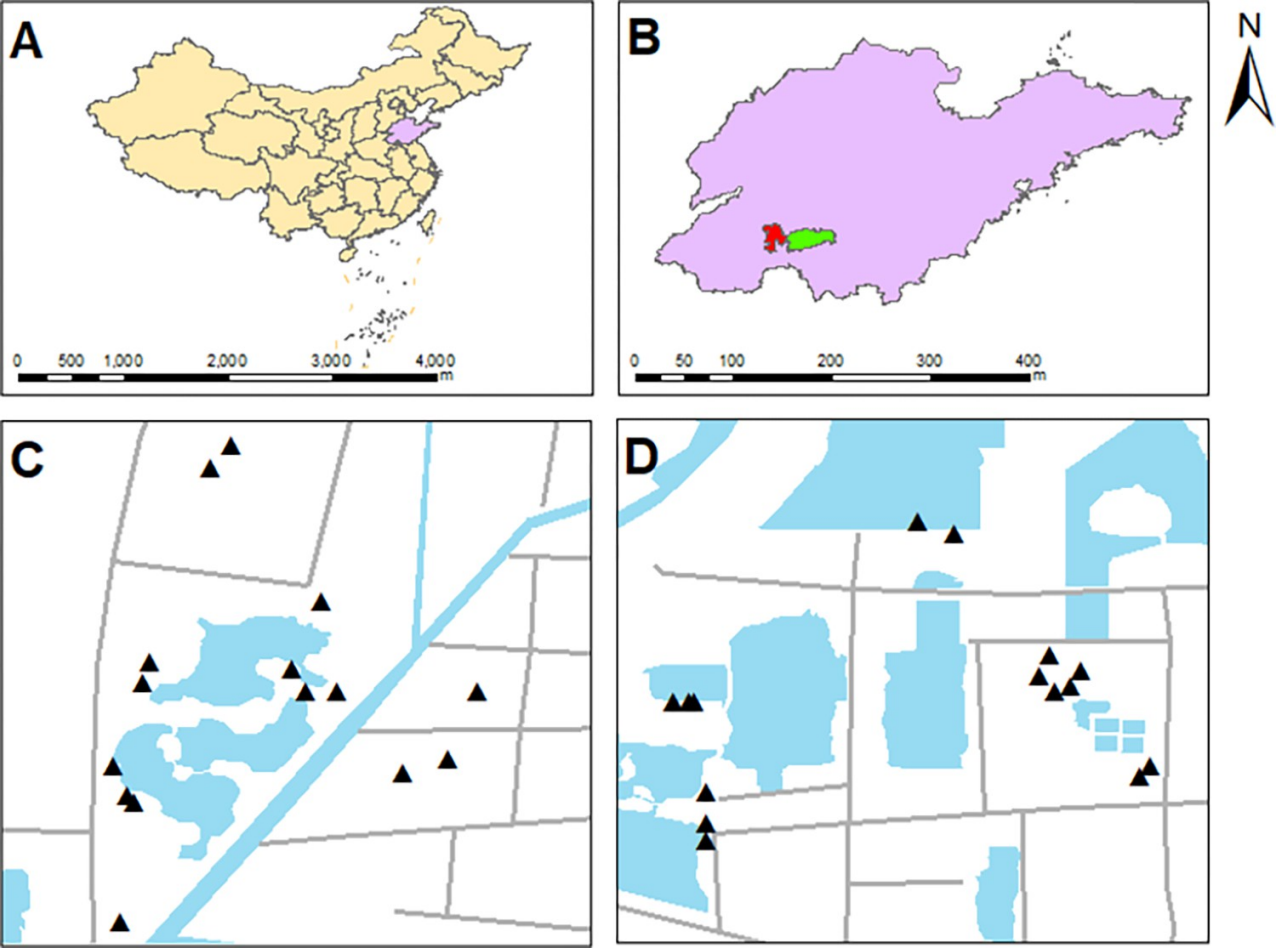
Research area and sample sites. Maps of Shandong Province in China (**A**) and parts of Jining City in Shandong Province (**B**) were approved by the Ministry of Natural Resources of China and the approval numbers are GS (2020) No.4630. **C** and **D** are maps of the sample sites, made by ArcMap 10.8.1.

In October 2021, we selected three habitats (wetland, lakeside grassland and farmland) in ZWP and JWP (Fig 1). We set five 1 m × 1 m sampling sites in each habitat. At each sampling site, using a five-spot sampling method, samples of 0–20 cm topsoil were collected with a soil sampler. Each park collected fifteen bags of soil samples. And geographical co-ordinate of each site was collected by an app named the Sky Map (Shandong) as well as GPS positioning. There was no farmland in JWP, so to complete the experimental design we selected farmland nearby as a substitute (JN). JN was restored in 2012 and less affected by coal mining subsidence than the farmland in ZWP (ZN). All samples were stored at 4°C for analysis of nitrogen and the different forms of SOC firstly, then samples were naturally air-dried and sieved to 2 mm for soil properties analysis. A total of 30 bags were collected from the research areas and all experiments were completed within two months.

### Soil properties measurements

To measure the soil water content (SWC) and bulk density (BD) of the soil, the aluminum specimen boxes were dried in an oven at 105°C until their weight was constant. To measure the soil pH, a pH meter (PHS-3E, Shanghai INESA Instrument Co., Ltd, China) was used at a 1:2.5 ratio of fresh soil to water. To measure the soil electrical conductivity (EC), an EC meter (DDS-307A, Shanghai INESA Instrument Co., Ltd, China) was used at a ratio of 5.0 mL water to 1 g soil. We used spectrophotometry to measure the soil cation exchange capacity (CEC). Soil samples for CEC analysis were ground and passed through a 0.149 mm sieve. Specifically, 3.5 g of sieved air-dried soil was placed in a 100 mL constant-weight polyvinyl test tube, then added 50 mL of [Co(NH_3_)_6_]Cl_3_ (1.66 cmol/L) and shaken for 1 h, keeping it in suspension. Finally, centrifuge at 4000 r/min for 10 min and complete the determination within 24 h.

### SOC and nitrogen measurements

To explore the impact of different habitats on SOC, we divided it into dissolved organic carbon (DOC) and particulate organic carbon (POC), depending on its solubility. POC can be further divided into light fraction OC (LFOC) and heavy fraction OC (HFOC) based on its density [32–34]. To assess the DOC content, we followed the method of Jones and Willett [35]. Specifically, 10.00 g of sieved fresh soil was placed in a 100 mL constant-weight polyvinyl test tube. The supernatant was extracted from the soil using KCl solution (0.5 mol/L) and filtered with a 0.45 μm filter membrane, then analyzed using a total organic carbon analyzer (TOC-5000, Shanghai Metash Instruments Co., Ltd, China). To measure soil HFOC, LFOC, heavy fraction organic nitrogen (HFON), light fraction organic nitrogen (LFON), 10.00 g of air-dried soil sample was placed in a constant 100 ml centrifuge tube; 40 mL of NaI (1.7 g/cm^3^) was added and carefully stirred into the solution with a glass rod. After the mixture had been separated in an ultrasonic shaker for 10 min, it was centrifuged at 4, 200 r/min for 10 min. The LFOC of the suspension in heavy liquid was poured into a 0.043 mm copper sieve and filtered, during which time it was rinsed with dilute hydrochloric acid to remove inorganic carbon. The above steps were repeated two to three times. The collected LFOC was rinsed with CaCl_2_ (0.01 mol/L) and tested with AgNO_3_ (without I^−^-reaction), then rinsed with water and tested with silver nitrate (without Cl^−^-reaction). Dilute hydrochloric acid was added to the HFOC at the bottom of the tube to remove inorganic carbon. Then, 0.01 mol/L CaCl_2_, was stirred in, and it was centrifuged for 10 min, before the following procedure: discard the supernatant, repeat five times, test with AgNO_3_ (without I^−^-reaction), rinse with water, test with AgNO_3_ (without Cl^−^-reaction), and then dry to constant. The soil LFON and HFON were extracted along with LFOC and HFOC extraction. Finally, we used an elemental analyzer (Unicube, Elementar, Germany) to measure the content of soil HFOC, LFOC, HFON and LFON. Total carbon (TC) and total nitrogen (TN) were measured using the air-dried soil sample passed through 0.149 mm sieve and soak in 0.5 M HCl for 2 h in order to remove carbonates, then measured with an elemental analyzer (Unicube, Elementar, Germany) [36]. To measure NO_3_^−^-N and NH_4_^+^-N, 10.00 g of sieved fresh soil was placed in a 100 mL centrifuge tube, with 40 mL of KCl (2.0 mol/L), and shaken at 200 r/min for 1 h. After standing for 2 h, the supernatant was extracted and filtered with a 0.45 μm filter membrane, and then measured with an automated wet chemistry analyzer–continuous flow analyzer (San series, Skalar Analytical B.V., The Netherlands).

### Statistical analysis

This experiment was based on field sampling using randomly selected samples. Thus, some indicators had large intra-group variation, resulting in their standard deviation being too high. Before statistical analyses, we performed the Shapiro–Wilk test to examine data normality and transform the data by certain methods if necessary [37]. We only transformed the data of the content of DOC by ranking of cases. We then processed the statistical analyses using the following three steps. The first step was to perform a two-way analysis of variance (ANOVA) to determine the effects of different parks and habitats on the distributions of DOC, LFOC and HFOC. In the second step, the effect on the distribution of SOC within each park was determined by the Kruskal–Wallis test, Spearman’s correlation analysis and stepwise multiple linear regression analysis. Finally, the differences between the two parks were compared with the independent sample *t* test. These tests were performed using SPSS v25.0, and the construction of graphs by ArcMap 10.8.1 and Origin 2021.

## Results

### Differences in environmental factors and SOC content

**Fig 2:**
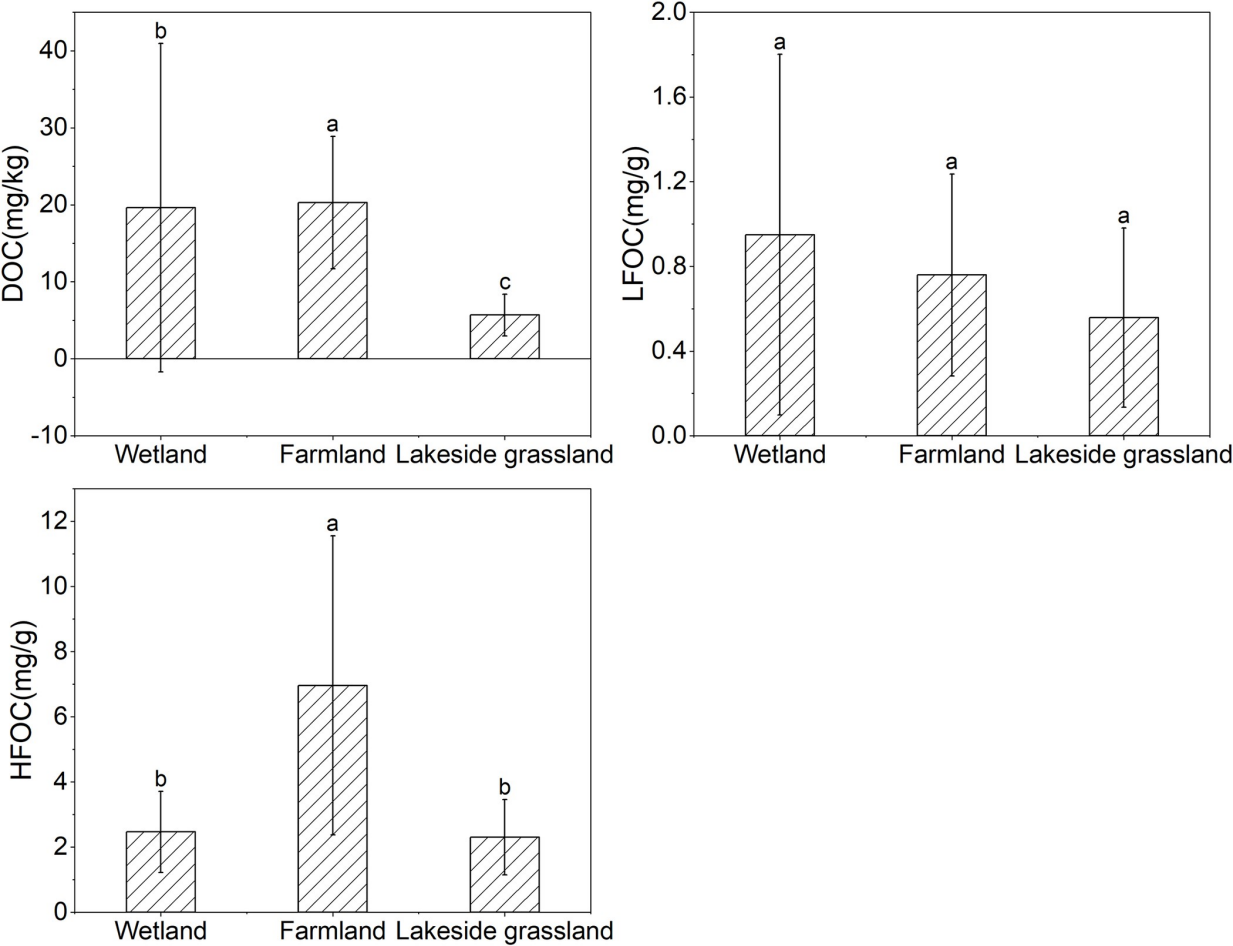
SOC content in different habitats. Error bars represent standard deviation of the mean (n = 5). DOC: dissolved organic carbon; LFOC: light fraction organic carbon; HFOC: heavy fraction organic carbon. Different lowercase letters indicate significant differences (*p* < 0.05).

**Fig 3:**
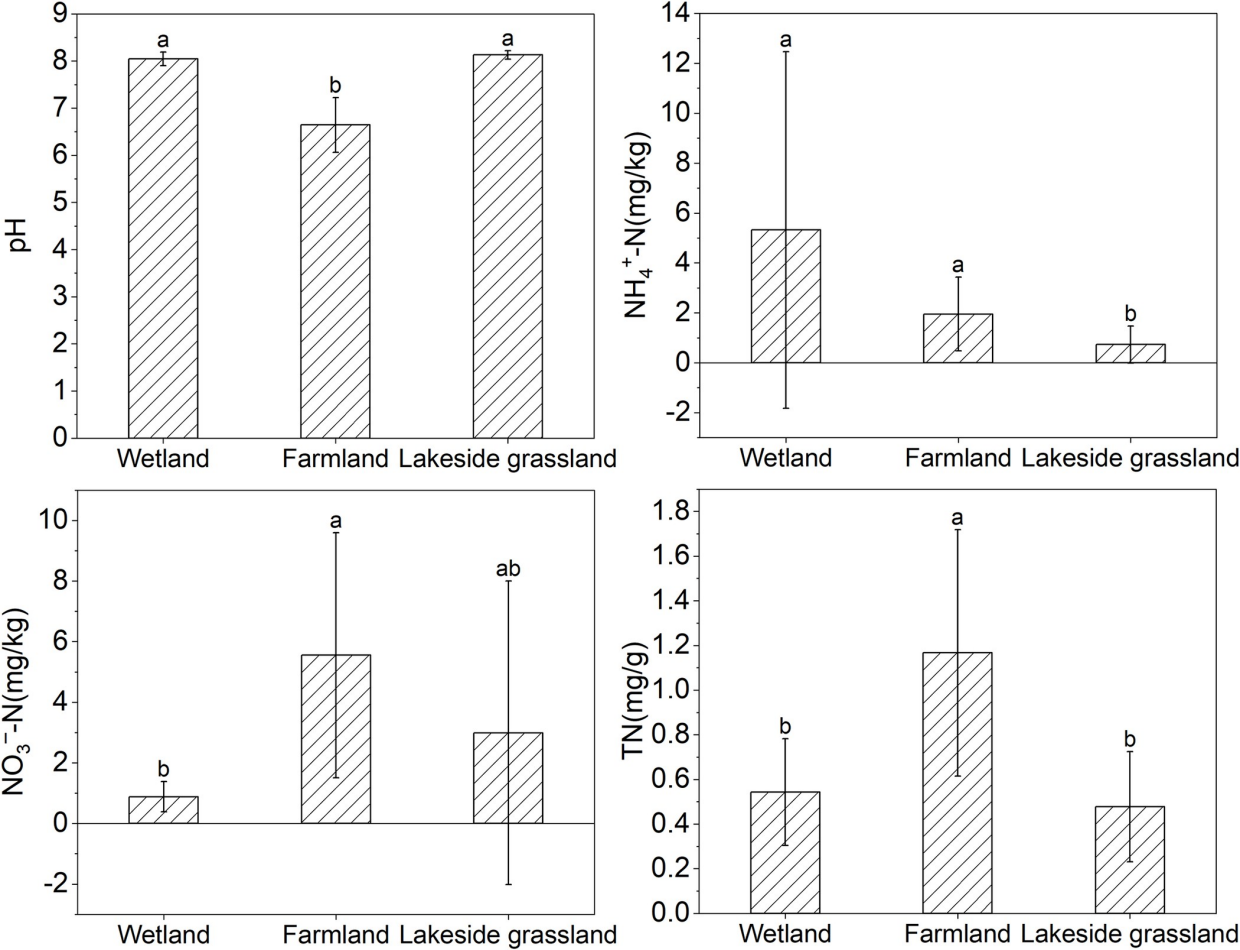
Soil properties in different habitats. Error bars represent standard deviation of the mean (n = 5). TN: total nitrogen; NO_3_^–^-N: nitrate nitrogen; NH_4_^+^-N: ammonium nitrogen. Different lowercase letters indicate significant differences (*p* < 0.05).

The two-way ANOVA showed that the contents of DOC and HFOC varied significantly in different habitats, indicating that habitats significantly altered the content and composition of SOC (*p* < 0.001; Table 1). The DOC content of the wetland (19.62 mg/kg) was significantly higher than that of the lakeside grassland (5.68 mg/kg), but significantly lower than that of the farmland (20.29 mg/kg). The HFOC content of the farmland (6.96 mg/g) was significantly higher than that of the lakeside grassland (2.31 mg/g) and wetland (2.47 mg/g; Fig 2). The Kruskal–Wallis test showed that the farmland pH (6.64) was significantly lower than that of the lakeside grassland (8.13) and wetland (8.04). The NH_4_^+^-N content of the lakeside grassland (0.73 mg/kg) was significantly lower than that of the wetland (5.32 mg/kg) and farmland (1.95 mg/kg), and the NO_3_^–^-N content of the farmland (5.55 mg/kg) was significantly higher than that of the wetland (0.88 mg/kg). The TN of the farmland (1.17 mg/kg) was significantly higher than the lakeside grassland (0.48 mg/kg) and wetland (0.54 mg/kg; Fig 3).

**Table 1:** ANOVA analysis of three forms of SOC.

	Wetland park	Habitat	Wetland park*Habitat
DOC	*NS*	***	*
LFOC	*NS*	*NS*	*NS*
HFOC	*NS*	***	**

Wetland park: Shili Lake Ecological Wetland Park (JWP) and Shandong Zoucheng Taiping National Wetland Park (ZWP). Habitat: farmland, wetland and lakeside grassland. DOC: dissolved organic carbon; LFOC: light fraction organic carbon; HFOC: heavy fraction organic carbon. *NS*: non-significant

*: *p* < 0.05

**: *p* < 0.01

***: *p* < 0.001.

Although SOC did not differ significantly between the wetland parks, DOC and HFOC contents were significantly different (*p* < 0.05) in the interaction between wetland park and habitat types.

### Soil properties and the content of SOC in JWP

**Fig 4:**
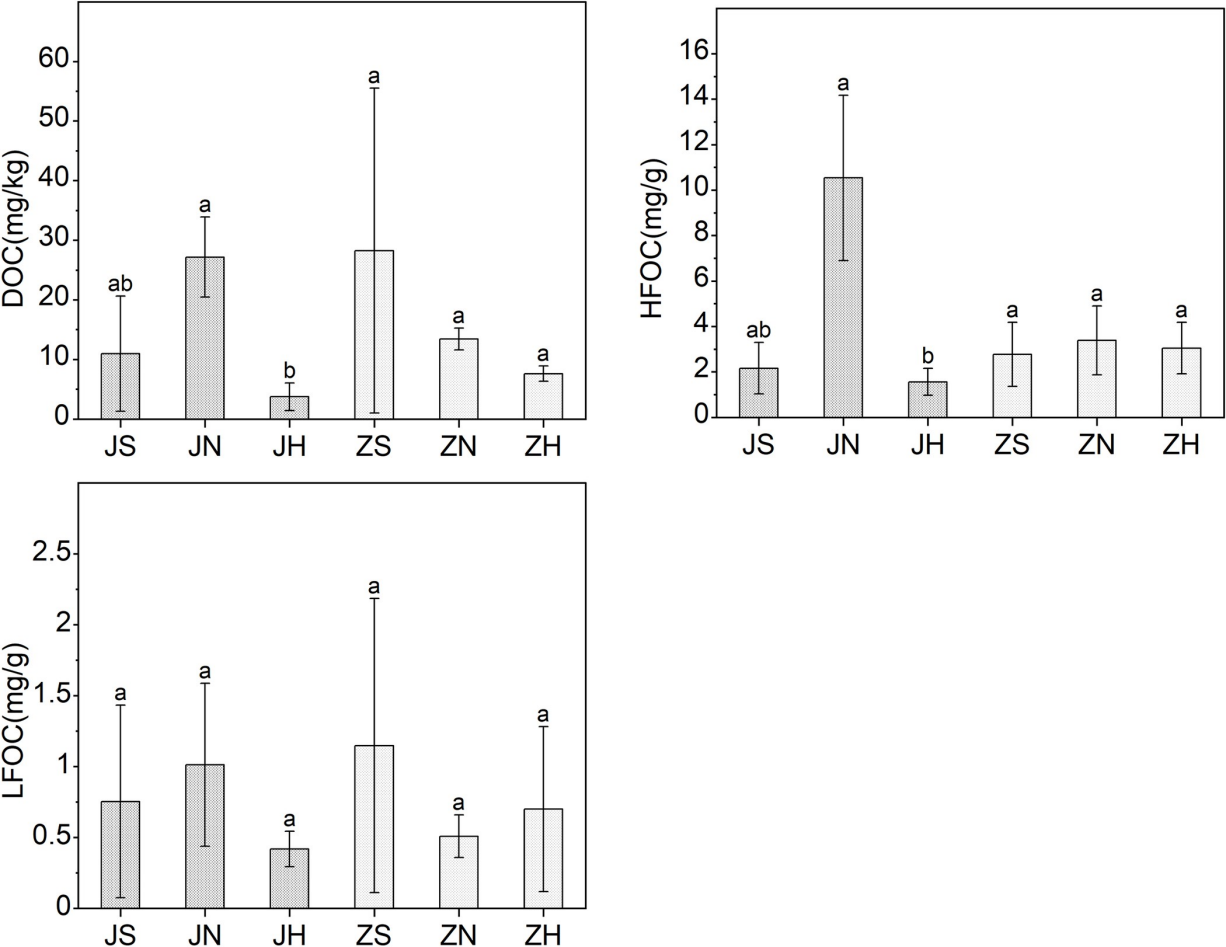
SOC comparison between different habitats in JWP and ZWP. JWP: Shili Lake Ecological Wetland Park; ZWP: Shandong Zoucheng Taiping National Wetland Park; JS, JN and JH are the wetland, farmland and lakeside grassland in JWP, respectively. ZS, ZN and ZH are the wetland, farmland and lakeside grassland in ZWP, respectively. DOC: dissolved organic carbon; LFOC: light fraction organic carbon; HFOC: heavy fraction organic carbon. Error bars represent standard deviation of the mean (n = 5). Different lowercase letters indicate significant differences (*p* < 0.05).

**Fig 5:**
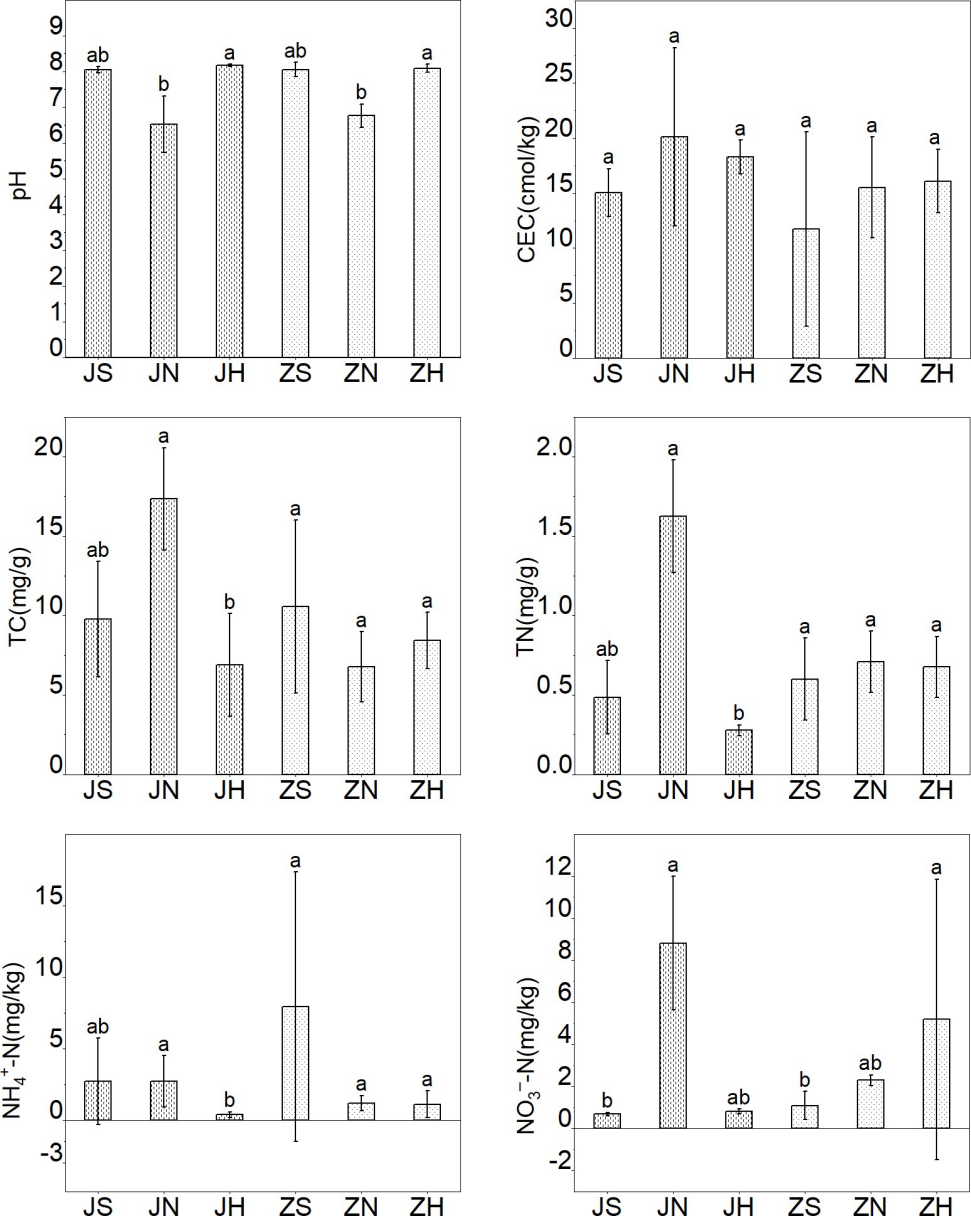
Soil properties in different habitats in JWP and ZWP. JWP: Shili Lake Ecological Wetland Park; ZWP: Shandong Zoucheng Taiping National Wetland Park Error bars represent standard deviation of the mean (n = 5). CEC: soil cation exchange capacity; TC: total carbon; TN: total nitrogen; NO_3_^–^-N: nitrate nitrogen; NH_4_^+^-N: ammonium nitrogen. Other abbreviations as in Fig 4. Different lowercase letters indicate significant differences (*p* < 0.05).

**Fig 6:**
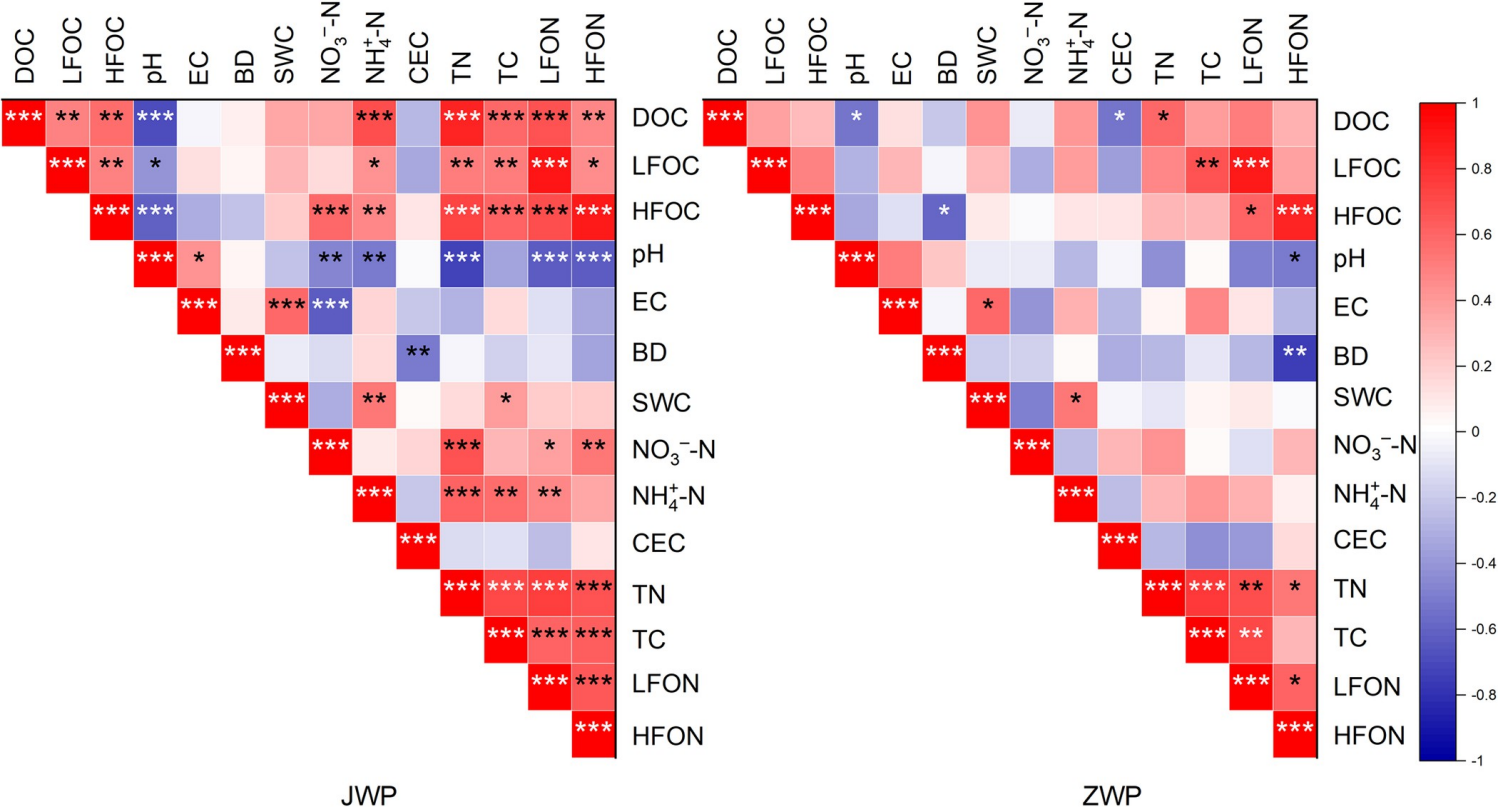
Spearman’s correlation analysis between SOC and various environmental factors in two wetland parks. JWP: Shili Lake Ecological Wetland Park; ZWP: Shandong Zoucheng Taiping National Wetland Park. Blue means negative correlation. Red mean positive correlation. The darker the color, the greater the correlation. EC: electrical conductivity; BD: soil bulk density; SWC: soil water content; LFON: light fraction organic nitrogen; HFON: heavy fraction organic nitrogen. Other abbreviations as in Figs 4 and 5. *: *p* < 0.05; **: *p* < 0.01; ***: *p* < 0.001.

In JWP, the DOC content (27.16 mg/kg) and HFOC content (10.54 mg/g) of the farmland were significantly higher than the DOC content (3.75 mg/kg) and HFOC content (1.56 mg/g) of the lakeside grassland (Fig 4). Soil properties were significantly different between the lakeside grassland and the farmland (Fig 5). Spearman’s correlation analysis showed that DOC content as well as HFOC was significantly correlated with pH and different forms of nitrogen.(Fig 6). Thus, it could be seen that the distributions of DOC and HFOC contents in JWP were closely related to the content and composition of nitrogen. LFOC did not show significant differences among the three habitats in JWP. A significant positive correlation between LFOC content and LFON content was found by Spearman’s correlation analysis, which was consistent with the results obtained for HFOC, indicating a significant coupling relationship between POC and nitrogen. Stepwise multiple linear regression showed that TN had effects on the content of DOC, HFOC, and LFOC. CEC also had effects on the content of LFOC (Table 2).

**Table 2:** Stepwise multiple linear regression for SOC in JWP.

DOC	Variable	Coef	LFOC	Variable	Coef	HFOC	Variable	Coef
R^2^ = 0.74	Constant	1.43	R^2^ = 0.68	Constant	1.79	R^2^ = 0.85	Constant	-0.54
	TN	15.75		CEC	-0.085		TN	6.65
				TN	0.564			

JWP: Shili Lake Ecological Wetland Park; Variable: the variables selected after stepwise multiple linear regression. Coef: the relationship between variable and SOC. R^2^: amount of variation in SOC explained. DOC: dissolved organic carbon; LFOC: light fraction organic carbon; HFOC: heavy fraction organic carbon. TN: total nitrogen; NO_3_^–^-N: nitrate nitrogen; NH_4_^+^-N: ammonium nitrogen; CEC: soil cation exchange capacity.

### Soil properties influencing the content of SOC in ZWP

In ZWP, there was no significant difference in SOC content among the three habitats (Fig 4). Spearman’s correlation analysis showed that DOC content was significantly correlated with pH, CEC and TN; HFOC content was significantly correlated with BD, LFON and HFON, among which it was significantly negatively correlated with BD (Fig 6). The results showed that soil basic properties had an effect on the distribution of organic carbon in ZWP. Stepwise multiple linear regression showed that the contents of NH_4_^+^-N, CEC, BD played an important role in the content of DOC. The content of NH_4_^+^-N had effects on the content of LFOC. The variation of the content of HFOC was lower explained by soil properties (Table 3).

**Table 3:** Stepwise multiple linear regression for SOC in ZWP.

DOC	Variable	Coef	LFOC	Variable	Coef	HFOC	Variable	Coef
R^2^ = 0.96	Constant	34.76	R^2^ = 0.68	Constant	0.502	R^2^ = 0.31	Constant	8.09
	NH_4_^+^-N	2.32		NH_4_^+^-N	0.083		BD	-4.00
	CEC	-0.69						
	BD	-12.96						

ZWP: Shandong Zoucheng Taiping National Wetland Park. Variable: the variables selected after stepwise multiple linear regression. Coef represents the relationship between variable and SOC. R^2^: amount of variation in SOC explained. DOC: dissolved organic carbon; LFOC: light fraction organic carbon; HFOC: heavy fraction organic carbon. NH_4_^+^-N: ammonium nitrogen; CEC: soil cation exchange capacity; BD: soil bulk density.

### Differences in SOC and environmental factors between JWP and ZWP

The independent sample *t* test showed that the differences between the two wetland parks were mainly in the farmland habitats. There were significant differences in NO_3_^–^-N and TN between the farmland and the lakeside grassland of the two parks. The only significant difference between the wetlands was NO_3_^–^-N content. Except for the farmland, the NO_3_^–^-N contents in the lakeside grassland and wetland were higher in ZWP than in JWP (Table 4).

**Table 4:** Differences in SOC and environmental factors in JWP and ZWP.

Habitat	Wetland park	DOC	HFOC	NO_3_^–^-N	TN
		mg/kg	mg/g	mg/kg	mg/g
Wetland	JWP	-	-	0.68±0.07	-
	ZWP	-	-	1.08±0.68	-
Farmland	JWP	27.16±6.73	10.54±3.64	8.81±3.18	1.62±0.36
	ZWP	13.41±1.83	3.39±1.51	2.29±0.26	0.71±0.19
Lakeside grassland	JWP	3.75±2.31	1.56±0.60	0.80±0.12	0.28±0.03
	ZWP	7.61±1.28	3.05±1.13	5.18±6.67	0.68±0.19

Values are expressed as mean ± standard deviations. JWP is Shili Lake Ecological Wetland Park. ZWP is Shandong Zoucheng Taiping National Wetland Park. DOC: dissolved organic carbon; HFOC: heavy fraction organic carbon; NO_3_^–^-N: nitrate nitrogen; TN: total nitrogen; Factors with no data show no significant differences between the two parks in the three habitats (*p* < 0.05).

## Discussion

### Differences in SOC content and composition between habitats

The DOC and HFOC contents of the farmland were significantly higher than the wetland and lakeside grassland. This may be because: (1) the tillage system of farmland was no-tillage and crop residues were left on the soil surfaces. A study has shown that soil organic matter (SOM), and even soil productivity, can be increased by maintaining surface residues through conservation tillage [10]; (2) no-tillage systems generally exhibit increased agglomerations and SOM compared to traditional farming [38]. Therefore, the sources of SOC and the physical protection of the farmland increased. At the same time, large aggregates were usually associated with unstable carbon and nitrogen [39]. And slower turnover of macroaggregates in no-tillage made more micro-aggregates formed in stable large aggregates than under conventional tillage, which were conducive to the consolidation of more crop-derived carbon [38]. (3) farmland had a higher content of nitrogen than the wetland and lakeside grassland due to nitrogen fertilizer. Many studies have shown that nitrogen restriction is widespread in many ecosystems [40–42]. Applying nitrogen fertilizer caused a large quantity of nitrogen to be added to the topsoil of farmland, which weakened the nitrogen limitation to some extent, thereby increasing the primary productivity of the plant [43]. Moreover, nitrogen enrichment may also have a negative impact on the growth of microorganisms, which may inhibit the decomposition of SOC by microorganisms, leading to SOC accumulation [41]. Therefore, the farmland could appear to accumulate a large quantity of DOC and HFOC. However, many studies have proved nitrogen fertilizer could decrease soil pH [41, 44, 45] and our analysis also came to the same conclusion. In general, the decrease of soil pH is not conductive to plant growth, but our study showed that the promotion of nitrogen addition to SOC accumulation played a major role in farmland. Meanwhile, a study showed that decreases in respiration rates of soil microbial were mainly a direct result of soil nitrogen availability increasing rather than indirect effects like the decreases of soil pH caused by nitrogen addition [46].

### Changes in habitats between different restoration time

Since the two wetland parks were built in different times, the restoration times of habitats were different. Except for the farmland, the restoration times of the wetland and lakeside grassland in ZWP were longer than those in JWP. Our analysis showed that even though the restoration times were different, the soil pH of the farmland was significantly lower than that of the wetland and lakeside grassland due to the long-term nitrogen fertilization. The relationship between carbon and nitrogen in the two wetland parks was strongly coupled, indicating that carbon and nitrogen had a strong correlation, consistent with previous study [47]. For example, the LFOC contents were found to be closely related to LFON and NO_3_^–^-N in both wetland parks. NO_3_^–^-N is one of the inorganic nitrogen sources needed for plant growth and is among the effective nitrogen sources that plants can directly use. LFOC and LFON were mainly derived from plant granular organic residues, meanwhile their turnover was faster which helped to provide phytonutrients, including NO_3_^–^-N [48]. A study has shown that the main determinants of soil carbon persistence differed between soil layers, and that plant carbon input was the main control of long-term soil carbon persistence in topsoil, which was the chief cause of SOM instability in this layer [49]. Therefore, the ratio of topsoil LFOC content to total SOM could provide an early and sensitive indicator of the consequences of different management measures [12]. The effects of microbial decomposition on carbon loss need a further analysis about the microorganisms. Through stepwise multiple linear analysis, we found that different composition of SOC in different parks responded differently to nitrogen. Specifically, the content of TN played an important role in JWP, while NH_4_^+^-N in ZWP. This phenomenon may be given that the different types of soil microorganisms in the two parks, resulting in different utilization of nitrogen sources. It also reflected the need for analysis of soil microorganisms.

Except for the wetland, there were significant differences between the content of DOC, HFOC and many soil properties in the farmland and lakeside grassland of the two wetland parks. Changes of soil properties in wetlands were not significant probably given that the recovery of the wetlands were slow, incomplete or even not be fully recovered over time [50]. In addition, there were significant differences in NO_3_^−^-N content between the three habitats. The likely reason was that, after remediation, microorganisms related to nitrification in the soil increased significantly over time. Recently, a study found that the responses of soil microorganisms under long-term nitrogen deposition were significantly different with different times [51]. Thus, it is necessary to find out how nitrogen, microorganisms and SOC interacted with each other over time which could better for us to understand the mechanisms of the accumulation of SOC.

The SOC contents in the wetland were found to be higher than in the lakeside grassland, but not significantly. Numerous studies have shown that natural wetlands have high SOC storage relative to other types of habitats [16, 52–55]. Wetland ecosystems release large quantities of carbon when they are destroyed or converted to other habitats [7], and it can be easy to overlook the fact that the conversion, degradation and warming of these systems could lead to rapid loss of carbon from ancient times [16]. The wetland habitats in this study failed to demonstrate their strong carbon sequestration potential, possibly due to the short restoration time of the two wetland parks selected. Degraded wetlands are thought to be able to recover their SOC storage functions through restoration [8], but it would take a long time.

## Conclusions

The study found that the DOC (20.29 mg/kg) and HFOC (6.96 mg/g) content of the farmland was significantly higher than that of the wetland (19.62 mg/kg, 2.47 mg/g) and lakeside grassland (5.68 mg/kg, 2.31 mg/g) generated by the restoration of coal mining subsidence land, due to nitrogen enrichment. Soil organic carbon had a strongly positive correlation with nitrogen enrichment, and the changes in microorganisms should be considered to better understand the mechanisms of the relationship between soil organic carbon and nitrogen. Moreover, through the comparison of the habitats with different restoration times, it was found that the farmland and with a longer restoration time had a stronger SOC storage capacity. In this study, the wetland habitats did not exhibit their ability to store OC, most likely because of the relatively short restoration time. In-depth analysis of changes in microorganisms over time may help us better explain these phenomena. Therefore, the SOC storage capacity of farmland destroyed by coal mining subsidence could be restored through ecological restoration, but the degree of recovery depends on the reconstructed habitat types and the restoration time.
